# The future of fundamental science led by generative closed-loop artificial intelligence

**DOI:** 10.3389/frai.2026.1678539

**Published:** 2026-02-11

**Authors:** Hector Zenil, Jesper Tegnér, Felipe S. Abrahão, Alexander Lavin, Vipin Kumar, Jeremy G. Frey, Adrian Weller, Larisa Soldatova, Alan R. Bundy, Nicholas R. Jennings, Koichi Takahashi, Lawrence Hunter, Saso Dzeroski, Andrew Briggs, Frederick D. Gregory, Carla P. Gomes, Jon Rowe, James Evans, Hiroaki Kitano, Ross King

**Affiliations:** 1 Research Departments of Biomedical Computing and Digital Twins, School of Biomedical Engineering and Imaging Sciences, King's College London, London, United Kingdom; 2 The Alan Turing Institute, British Library, London, United Kingdom; 3 Oxford Immune Algorithmics, London Institute for Healthcare Engineering, Oxford University Innovation, London, United Kingdom; 4 Algorithmic Dynamics Lab, King's Institute for AI, King's College London, London, United Kingdom; 5 Department of Chemical Engineering and Biotechnology, University of Cambridge, Cambridge, United Kingdom; 6 Cancer Research Group, The Francis Crick Institute, London, United Kingdom; 7 Living Systems Laboratory, BESE, CEMSE, King Abdullah University of Sciences and Technology, Thuwal, Saudi Arabia; 8 Department of Medicine, Karolinska Institutet, Stockholm, Sweden; 9 Centre for Logic, Epistemology and the History of Science, University of Campinas, Campinas, São Paulo, Brazil; 10 DEXL, National Laboratory for Scientific Computing, Rio de Janeiro, Brazil; 11 Pasteur Labs, New York, NY, United States; 12 Institute for Simulation Intelligence, New York, NY, United States; 13 Department of Computer Science and Engineering, University of Minnesota, Minneapolis, MN, United States; 14 Department of Chemistry, University of Southampton, Southampton, United Kingdom; 15 Department of Computing, Goldsmiths, University of London, London, United Kingdom; 16 School of Informatics at the University of Edinburgh, Edinburgh, United Kingdom; 17 Vice-Chancellor's Office, Loughborough University, Loughborough, United Kingdom; 18 RIKEN Center for Biosystems Dynamics Research, Tokyo, Japan; 19 RIKEN Innovation Design Office, Tokyo, Japan; 20 Keio University, Tokyo, Japan; 21 Center for Computational Pharmacology, School of Medicine, University of Colorado, Aurora, CO, United States; 22 Department of Knowledge Technologies, Jozef Stefan Institute, Ljubljana, Slovenia; 23 Department of Materials, University of Oxford, Oxford, United Kingdom; 24 DEVCOM ARL Army Research Office, London, United Kingdom; 25 Department of Computer Science, Cornell University, Ithaca, NY, United States; 26 School of Computer Science, University of Birmingham, Birmingham, United Kingdom; 27 Knowledge Lab, University of Chicago, Chicago, IL, United States; 28 The Systems Biology Institute, Okinawa Institute of Science and Technology, Naha, Japan; 29 Chalmers Institute of Technology, Gothenburg, Sweden

**Keywords:** AI4Science, AI-conducted science, closed-loop discovery, cognitive collapse, domain-method alignment, epistemic singularity, graded autonomy, human-machine collaboration

## Abstract

Artificial intelligence is approaching the point at which it can complete the scientific cycle, from hypothesis generation to experimental design and validation, within a closed loop that requires little human intervention. Yet, the loop is not fully autonomous: humans still curate data, set hyperparameters, adjudicate interpretability, and decide what counts as a satisfactory explanation. As models scale, they begin to explore regions of hypothesis and solution space that are inaccessible to human reasoning because they are too intricate or alien to our intuitions. Scientists may soon rely on AI strategies they do not fully understand, trusting goals and empirical payoffs rather than derivations. This prospect forces a choice about how much control to relinquish to accelerate discovery while keeping outputs human relevant. The answer cannot be a blanket policy to deploy LLMs or any single paradigm everywhere. It demands principled matching of methods to domains, hybrid causal and neurosymbolic scaffolds around generative models, and governance that preserves plurality and counters recursive bias. Otherwise, recursive training and uncritical reuse risk model collapse in AI and an epistemic collapse in science, as statistical inertia amplifies flaws and narrows the investigation. We argue for graded autonomy in AI-conducted science: systems that can close the loop at machine speed, while remaining anchored to human priorities, verifiable mechanisms, and domain-appropriate forms of understanding.

## 1 Introduction

**Figure 1:**
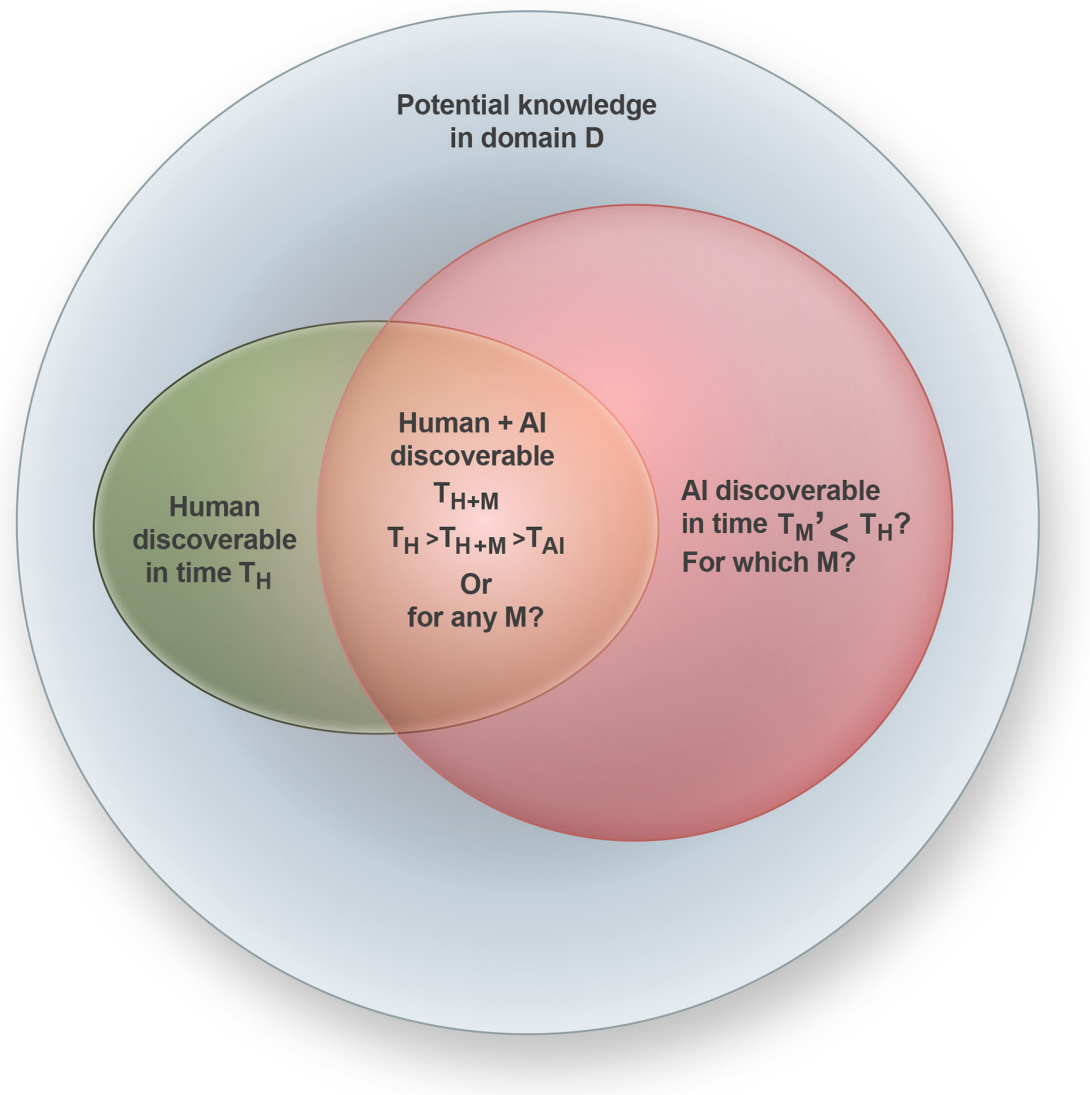
A quantitative framework of domain-dependent (D) and domain-agnostic acceleration of scientific discovery with AI, its relationship with human-carried science, and the combination of human and machine is yet to be explored more deeply. AI time *T*_*M*_ to conduct certain tasks is traditionally taken to be faster than *T*_*H*_ by orders of magnitude (King et al., 2004) as it is also more scalable. Still, its domain-dependency and relationship to *T*_*H*+*M*_ (human-machine hybrid) are likely highly domain-specific and has traditionally ignored closed-loopness or the removal of any human input.

With the scientific revolution in the seventeenth century, mathematical modeling through equations became the most efficient language for understanding and predicting events in the natural world. Four centuries later, we possess unprecedented volumes of data and access to vast computational resources. In recent years, the comprehensive application of machine learning has accelerated science in ways previously thought impossible, raising questions about how to quantify the rate of discovery (Figure 1). One consequence of this digital acceleration is that it has become increasingly difficult for individual scientists to follow developments across their fields and assimilate the growing volume of literature. To advance science and perform end-to-end high-quality investigations, scientists often require one or two orders of magnitude more hypothesis-led experiments than are currently humanly possible. Laboratories are under pressure to perform an ever-increasing number of experiments to replicate and validate results, making collaborations more complex, particularly across disciplines. It may be that certain frontiers of science, such as new fundamental physics or biological theory, will soon be too difficult for humans to advance unaided. AI systems may therefore need to take the lead in knowledge discovery if science is to sustain its momentum.

Recent advances in AI since 2010 (LeCun et al., 2015; Schmidhuber, 2015), fuelled by large datasets and computing power, have primarily addressed pattern recognition, object classification, and game playing. Following this period of opaque and difficult-to-interpret “black-box” models, there has been renewed interest in extending the scope of AI toward more transparent and scientifically grounded approaches. These include drawing inspiration from neuroscience (Hassabis et al., 2017; Zador et al., 2023), learning causality (Pearl, 2009), incorporating geometric and physical priors in model design (Bronstein et al., 2017; Karniadakis et al., 2021; Lavin et al., 2021), and developing explanatory and interpretable machine learning (Holzinger et al., 2022). A distinct research community has emerged under the banner of AI4Science (AI for Science, 2023), now active across numerous disciplines (Berens et al., 2023; Karagiorgi et al., 2022; Raghu and Schmidt, 2020; Noé et al., 2020; Richards et al., 2019), focusing on data integration, workflow automation, parameter optimisation, and scientific visualization (Wang et al., 2023). Most recently, the development of foundational transformer models (Bommasani et al., 2022; OpenAI, 2023) has given rise to the idea that, given sufficient data, such systems might acquire emergent understanding of their domains (Srivastava et al., 2023). Whether this requires intelligent priors or merely scale remains unresolved. In either case, there remains a substantial gap between these advances and the creation of an artificial scientist capable of autonomously formulating new scientific laws. In this perspective, we propose that a closed-loop iterative formulation offers a concrete pathway toward that objective.

Closing the scientific loop, however, may also accelerate an epistemological rupture. Once AI systems are allowed to design, conduct, and interpret experiments with minimal human supervision, they will begin to explore regions of the hypothesis and solution space that are inaccessible or unintuitive to human reasoning. At that point, science itself may start to evolve according to logics that humans can exploit but no longer fully understand. The phenomenon resembles Magnus Carlsen's evolution in chess after studying AlphaZero's games. Inspired by the engine's “alien” strategies, characterized by aggressive h-pawn advances, early rook mobilization, and long-term positional sacrifices, Carlsen developed a style that departed from classical chess theory yet yielded superior control. He learned not the method but the goal, playing for dynamic advantage without reconstructing the underlying rationale. Closed-loop science may similarly lead researchers to depend on AI systems that achieve empirically valid results through reasoning opaque to human interpretation. The shift from comprehension to instrumental trust could thus become the defining feature of AI-conducted discovery.

The challenge is to decide how far this shift should proceed. Total automation might allow progress at machine speed, yet it risks detaching science from human relevance and interpretability. Conversely, retaining humans in every loop may constrain exploration to familiar regions of the hypothesis space. Striking a balance requires principled alignment between methods and domains rather than uniform adoption of any single architecture. Large Language Models, for example, exhibit statistical inertia when trained recursively on their own outputs, leading to degenerative feedback or model collapse. Should such feedback propagate into scientific discovery pipelines, the consequence would not merely be the degradation of models but a broader epistemic collapse, in which science itself becomes self-referential and narrow. Avoiding this outcome demands hybrid architectures and domain-sensitive frameworks that integrate generative, causal, and neurosymbolic reasoning within the closed loop, maintaining both efficiency and explanatory diversity.

To conceptualize how AI can augment science, we distinguish three levels of operation. First, AI can act as an extractor of information, for example by mining the scientific literature and synthesizing knowledge from vast sources. Second, AI can make existing workflows more efficient by automating simulations and analyses or integrating multiple parameter-dependent steps into a single optimisation process, as in the first version of AlphaFold (Senior et al., 2020). At these two levels, AI primarily enhances rather than replaces human practice. The third level, which forms the focus of this work, envisions AI systems capable of discovering new laws or representations of nature. These may arise through latent representations learned directly from data or through models constrained by human-defined priors enforcing symmetries and invariants. Geometric machine learning continues this classical model-based reasoning that began with the scientific revolution. The challenge is to identify such useful priors directly from data rather than imposing them externally. Here we focus on how a closed-loop AI-science cycle could enable such interpretable representations to emerge autonomously.

AI can accelerate the entire scientific process while simultaneously advancing AI itself in domains relevant to fundamental science, including causal discovery, automated experimentation, and knowledge expansion. One persistent bottleneck is knowledge representation, which remains biased toward human conceptual frameworks. A fully closed-loop science-AI system integrated with laboratory automation could, in principle, execute complete experimental cycles (King et al., 2004, 2009). However, the consequences of closing the loop remain uncertain (Figure 1). Although such systems can already automate simple scientific tasks, the overall productivity of human-AI collaborations compared to fully autonomous AI remains unknown. Hybrid systems may yield outcomes that are both more productive and more aligned with human interests. Yet restricting AI to human comprehensibility may also prevent exploration of hypothesis spaces that, while initially unintuitive, could lead to major breakthroughs. Whether partnering with AI or allowing it greater autonomy will enable science to cover a larger portion of potential human knowledge—defined recursively as the knowledge that could, in principle, be rendered intelligible—remains an open question.

So far, AI's most prominent successes have occurred in well-bounded domains with clear metrics of performance (McCarthy, 2007), such as games and bioinformatics (Silver et al., 2018; Stokes et al., 2020; Alipanahi et al., 2015; Senior et al., 2020). These achievements rely on powerful search algorithms and efficient end-to-end workflows, yet they have not produced new fundamental laws of nature. To move beyond this stage, AI must develop the ability to discover transparent and generative representations of scientific phenomena. The concept of an iterative closed-loop discovery scheme offers one plausible path forward, though only a few examples have closed the full loop of discovery (King, 2011). Scientists must therefore design AI systems capable of sharing responsibility for observation, hypothesis generation, experimentation, evaluation, and integration of results into existing knowledge (Wang et al., 2019). The objective is not merely to accelerate discovery but to improve the reliability, reproducibility, and transparency of science as it becomes increasingly automated.

Figure 1 presents a framework for evaluating how domain-dependent and domain-agnostic AI methods may accelerate scientific discovery. While the main expectation is that AI will deliver substantial gains across most domains, the nature and scale of these gains hinge on temporal complexity. Although the machine timescale *T*_*M*_ is conventionally considered to be much shorter than the human timescale *T*_*H*_ (King et al., 2004), this assumption overlooks the intricacies of closed-loop discovery systems. When learning, experimentation, and model refinement are embedded within the loop, the effective rate of discovery may not scale linearly with computational speed. Bottlenecks in validation, interpretability, and human-AI coordination can dominate the cycle. A central open question is therefore whether hybrid human-machine systems achieve *T*_*H*+*M*_ < min(*T*_*H*_, *T*_*M*_), that is, whether teaming up truly outperforms both human-only and AI-only workflows. Evidence from radiology suggests that this is not always the case: A multisite study involving 140 radiologists on 15 chest radiographic tasks found that AI assistance improved performance for some but degraded it for others, depending on individual and contextual factors (Pesheva, 2024). Similarly, an experiment in mammography interpretation showed that even experienced radiologists suffered a drop in diagnostic precision from 82% to 45.5% when an AI system provided incorrect suggestions, illustrating how automation bias can reduce both accuracy and efficiency (Dratsch et al., 2023). These findings indicate that merely inserting humans into the loop does not guarantee acceleration or robustness. The key question is therefore not whether to automate or not, but which stages of the scientific process—hypothesis generation, experimentation, validation, or synthesis—benefit from human-AI collaboration, and under what conditions. Addressing this requires fine-grained quantification of discovery time, interaction overhead, and error propagation across these stages. Only through such measurement can we determine when hybrid closed-loop systems genuinely accelerate discovery and when they instead introduce new epistemic and temporal bottlenecks.

## 2 AI in scientific discovery

### 2.1 Historical context

From the seventeenth-century scientific revolution onwards, mathematical formalism became the principal language of explanation in natural philosophy. Newton's laws and the rise of analytical mechanics established a tradition of describing nature through differential equations, giving rise to a mode of discovery grounded in symbolic manipulation and deductive reasoning. The nineteenth and twentieth centuries added further layers of abstraction: probability theory, thermodynamics, and statistical mechanics introduced stochasticity into physical law, while computation and digital simulation extended mathematics to systems too complex for closed-form analysis. By the late twentieth century, data-driven statistical inference had become a dominant force in empirical science, transforming genomics, astronomy, and particle physics alike.

The early twenty-first century introduced a new epistemic transformation. Vast increases in data availability and computational power ushered in the algorithmic era, where machine learning became both microscope and telescope for pattern discovery. In this historical continuum, closed-loop AI systems represent the next inflection point: the first moment when hypothesis generation, experimental design, and evaluation can be linked within a self-correcting feedback system. This shift, from descriptive equations to autonomous inference, signals not a replacement of scientific reasoning but a reconfiguration of it—an expansion of epistemic scope, where AI participates as both instrument and interlocutor in the production of knowledge.

### 2.2 From automation to autonomous discovery

Artificial intelligence has long supported the scientific enterprise through automation, data analysis, and simulation. Its traditional role was instrumental—enhancing efficiency, scaling experimentation, and mitigating human error. Now, AI is transitioning from passive assistance to active participation in discovery. Modern AI systems can process information at scales and speeds that exceed human limits, detecting latent regularities and potential causal structures across vast datasets. This shift has given rise to *AI4Science*, a rapidly expanding domain integrating learning algorithms into every stage of inquiry (AI for Science, 2023; Berens et al., 2023; Karagiorgi et al., 2022; Raghu and Schmidt, 2020; Noé et al., 2020; Richards et al., 2019). Its approaches span statistical, symbolic, generative, causal, and algorithmic paradigms, all converging on the same goal: to accelerate and deepen the cycle of hypothesis formation, experimentation, and validation.

The first generation of AI-driven science largely focused on automation—enhancing measurement precision, parameter optimisation, and computational throughput (Wang et al., 2023). AlphaFold, for instance, demonstrated that end-to-end architectures could compress multi-step workflows into a single differentiable optimisation pipeline, revolutionizing protein structure prediction (Senior et al., 2020). Yet, while transformative, these systems do not yet alter the epistemic foundations of science. They expedite established procedures but remain anchored to human-defined objectives, loss functions, and validation standards. They advance performance without necessarily expanding understanding.

A second generation of scientific AI now extends beyond optimisation toward hypothesis generation and theory refinement. These models learn invariant structures, symmetries, or conserved quantities directly from data (Bronstein et al., 2017; Karniadakis et al., 2021). Neurosymbolic and causal frameworks embed scientific priors within learning architectures, allowing systems not only to predict but also to explain (Pearl, 2009; Holzinger et al., 2022). Closed-loop configurations take this one step further by coupling experiment design, data acquisition, and model updating within continuous feedback, creating systems that autonomously refine both their hypotheses and their strategies. As illustrated in Figure 1, such feedback loops could, in principle, compress discovery timescales by orders of magnitude, although their realized benefit remains highly domain-dependent.

### 2.3 The closed loop and the human—Machine boundary

Closing the scientific loop—where observation, hypothesis generation, and validation occur autonomously—marks a profound epistemological shift. Once AI systems can design and interpret experiments independently, they may begin to explore hypothesis spaces inaccessible to human reasoning. At this point, scientific inquiry risks diverging from human intuitions about causality and explanation. This echoes Magnus Carlsen's adoption of AlphaZero inspired strategies in chess: a style defined by exploiting goal-orientated patterns that are empirically dominant, but conceptually opaque. The human can exploit the method's outcomes without fully grasping its rationale. Scientific AI may soon reach a similar state, yielding empirically valid yet conceptually alien insights. The core challenge becomes deciding how much epistemic control humans should retain to keep discovery aligned with human relevance, without throttling the creative potential of machine-led inference.

Empirical evidence from hybrid human-AI systems reveals how non-trivial this trade-off is. In radiology, for instance, collaborative teams of humans and AI have not always outperformed either component alone. A large multi-site study involving 140 radiologists across 15 chest X-ray tasks found that AI assistance improved performance for some participants but degraded it for others, depending on context and experience (Pesheva, 2024). Similarly, in mammography interpretation, experienced radiologists suffered a reduction in diagnostic accuracy—from 82% to 45.5%—when AI provided misleading cues, a manifestation of automation bias (Dratsch et al., 2023). Such results underline that collaboration must be designed, not assumed: feedback, interpretability, and calibration mechanisms must be explicit for the hybrid to be more than the sum of its parts.

### 2.4 Graded autonomy and efficiency

The trajectory of AI in science will likely evolve through graded autonomy—an adaptive continuum where machines act independently in data-rich, low-risk domains but maintain human interpretive oversight in others. The division of labor between human and machine will shift dynamically as both scientific complexity and AI sophistication coevolve. A key objective will be quantifying when automation ceases to accelerate discovery, identifying thresholds where coordination, interpretability, or validation costs outweigh computational gains.

To reason about this formally, consider a recursive closed-loop cycle with total time complexity


$$T^{\prime }=T\left(T_{\text{obs}}+T_{\text{hyp}}+T_{\text{exp}}+T_{\text{eval}}+T_{\text{int}}\right),$$

where each component represents the duration of observation, hypothesis generation, experimentation, evaluation, and integration into the scientific knowledge. Then the whole process can iterate again in an infinite open-ended loop as it happens with human practice of science. Human-led science operates on the timescale *T*_*H*_, while a fully automated system operates on *T*_*M*_, typically orders of magnitude shorter for data acquisition and model evaluation (King et al., 2004). In hybrid systems, interaction overhead introduces an additional coordination term *C*_int_, giving


$$T_{H+M}=T_{H}+T_{M}+C_{\text{int}}.$$

This term is not only logistical but cognitive, whether the combination is detrimental or additive but it is most likely where the human cognitive load will operate and happen. Whether hybrid systems outperform either endpoint will depend critically on how *C*_int_ scales with task complexity and epistemic load. In highly automated domains such as materials design, for example, *C*_int_ → 0, may produce exponential speed-ups if little oversight is needed, letting the system explore the space of new AI-discoverable materials. In interpretive domains such as medicine or social science, however, *C*_int_ may dominate the loop, turning potential acceleration into drag. The grand challenge is thus to discover when *T*_*H*+*M*_ < min(*T*_*H*_, *T*_*M*_)—when collaboration truly outpaces either actor alone—and to establish principled frameworks for measuring epistemic and temporal efficiency. This is an open question that we believe will be highly domain-dependent and will require empirical advancements and trial and error before being answered but it will dominate the future.

Scientific discovery can be conceptualized as a self-referential process comprising five iterative phases: observation, hypothesis generation, experimentation, evaluation, and reintegration of results (positive or negative) into the body of knowledge. Each phase operates at distinct levels of abstraction, timescale, and interpretability. The goal of closed-loop architectures is to connect these stages dynamically so that models, hypotheses, and experiments co-evolve until convergence.

Table 1 maps scientific domains against the algorithmic classes of AI that best complement their epistemic structure. The table is not prescriptive but diagnostic: it reveals which methodologies align naturally with the ontological and data characteristics of each field. Reinforcement learning and evolutionary strategies are suited to domains with delayed rewards and high-throughput experimentation, such as drug discovery or materials synthesis. Neurosymbolic and structural-causal models are better aligned with theory-building disciplines requiring mechanistic interpretability and generalization. Algorithmic Information Dynamics offers tools for uncovering generative mechanisms in systems with discrete causal architectures, while supervised kernel-based learning thrives in data-rich empirical contexts with clear labels and noise models. LLMs, however, have opened AI to all fields massively by breaking into human language underpinning human science suddenly becoming applicable in all domains.

**Table 1:** Scientific domains clustered by domain sharing same color, mapped with most suitable list of AI algorithm classes and approaches.

**Domains**	**Large language models (LLMs)**	**Supervised—NN andkernel-based**	**Supervised—probabilisticand rules-based**	**Semi-supervised**	**Generative/diffusion models**	**Unsupervised**	**Algorithmic information dynamics**	**Soft computing and probabilistic numerics**	**Expert systems**	**Model-based**	**Structural causal**	**Neurosymbolic**	**Reinforcement learning**	**Neuroevolution**	**Genetic algorithm**	**Open-ended search, evolution**	**Quantum amplitudes andGrover search**	**QM Markov chains andsimulation**	**Data-driven, supervised quantum (NNs)**
Mathematics	✓	-	~	-	~	-	✓	-	✓	-	-	~	~	-	-	-	-	-	-
HE Physics - theo	✓	-	~	-	~	-	~	-	✓	-	~	~	-	-	-	~	~	✓	-
HE Physics - exp	✓	✓	✓	✓	✓	-	-	✓	-	✓	~	~	✓	~	-	-	✓	✓	✓
Optics & Acoustics	✓	✓	✓	~	✓	~	~	✓	-	~	-	~	~	-	~	-	~	-	-
Complexity	✓	-	-	~	✓	~	✓	-	✓	✓	✓	~	✓	~	✓	✓	-	~	-
SynBio & Ind Biotech	✓	✓	✓	~	✓	✓	~	~	✓	✓	~	~	✓	~	✓	~	-	~	~
Organic Chemistry	✓	✓	✓	✓	✓	✓	✓	✓	✓	✓	✓	✓	✓	✓	✓	✓	✓	✓	✓
Physical Chemistry	✓	✓	✓	✓	-	-	-	-	-	-	-	-	-	-	-	-	-	-	-
Electrochemistry	✓	✓	✓	-	-	-	-	-	-	-	-	-	-	-	-	-	-	-	-
Materials	✓	✓	✓	~	✓	~	-	-	✓	-	~	~	✓	~	~	~	-	~	~
Computing	✓	✓	✓	-	✓	-	-	~	-	-	-	~	✓	~	✓	-	~	✓	✓
Medicine, molecules/proteins	✓	✓	✓	-	~	-	~	-	~	~	~	✓	~	✓	~	-	-	~	-
Medicine, drug development	✓	✓	✓	~	~	-	✓	~	✓	✓	✓	✓	✓	~	~	-	-	-	-
Medicine, clinical	✓	✓	✓	-	✓	-	~	~	✓	✓	✓	~	~	-	-	-	-	-	-
Botany and zoology	✓	✓	✓	~	~	~	~	-	-	-	~	-	-	-	✓	✓	-	-	-
Systems bio and epidemiology	✓	~	✓	~	✓	-	~	✓	✓	✓	✓	✓	✓	~	✓	✓	-	~	-
Neuro and cog sciences	✓	✓	✓	~	✓	~	~	✓	-	✓	~	✓	✓	~	~	~	~	✓	~
Energy—nuclear (fis/fus)ion	✓	✓	✓	-	~	-	~	~	✓	✓	~	~	✓	~	-	~	~	~	~
Energy, generation and storage	✓	✓	✓	-	✓	-	~	~	✓	✓	~	-	✓	~	✓	~	~	-	~
Energy, oil and gas	✓	✓	✓	~	-	-	-	-	✓	~	~	-	✓	-	-	~	-	-	-
Manufacturing	✓	✓	✓	-	-	~	-	-	✓	-	-	~	~	-	~	-	-	-	-
Engineering and industrials	✓	✓	✓	-	~	✓	-	~	✓	✓	~	~	✓	-	-	~	-	-	~
Energy systems	✓	✓	✓	-	~	✓	-	✓	✓	✓	~	~	~	-	-	~	-	-	-
Transp. and infrastructure	✓	~	✓	-	-	~	-	-	✓	✓	~	-	~	-	-	~	-	-	-
Agriculture	✓	~	✓	~	✓	~	-	~	✓	✓	✓	~	✓	-	~	~	-	-	-
Ecology	✓	✓	✓	~	✓	~	-	~	✓	✓	✓	~	~	~	✓	✓	-	-	-
Socioeconomics and markets	✓	✓	✓	~	-	-	~	~	✓	✓	~	~	✓	~	✓	~	-	-	-
Finance	✓	✓	✓	-	-	-	-	-	✓	✓	~	~	✓	-	✓	-	-	-	-
Politics and geopolitics	✓	-	~	-	~	~	-	~	✓	~	~	-	~	-	~	~	-	-	-
Defense, aerospace	✓	✓	✓	✓	-	-	✓	✓	✓	~	~	~	~	~	~	-	-	-	-
Climate, weather	✓	~	✓	✓	✓	~	-	-	~	✓	~	✓	~	~	✓	✓	-	~	~
Earth systems	✓	~	✓	~	~	~	~	~	~	✓	~	✓	~	~	~	~	-	~	-
Astrophysics and cosmology	✓	✓	✓	✓	✓	✓	✓	-	-	~	~	-	-	~	~	~	~	~	~
Philosophy, epistemology	✓	-	-	-	-	-	✓	-	~	-	~	-	-	-	~	✓	-	-	-

LLMs have made AI universally applicable by hacking into the human scientific natural language. Markers: “-” and ✓mean no (or unknown) and yes, respectively; ~ implies the ML application is likely but not generalized.

Ultimately, the purpose of closed-loop AI science is not to remove humans from the process, but to reassign cognitive responsibility (e.g. Abdel-Rehim et al., 2025), improve and accelerate outcomes. By quantifying *T*_*H*_, *T*_*M*_, and *C*_int_, we can begin to chart where automation truly augments human performance and where it risks epistemic collapse. LLMs make *C*_int_ even more relevant or tangible because it is through natural human language that *C*_int_ may take its most common form, capturing the time humans may be able to interpret AI results by way of its most expressive current and intuitive human-AI interface.

**Figure 2:**
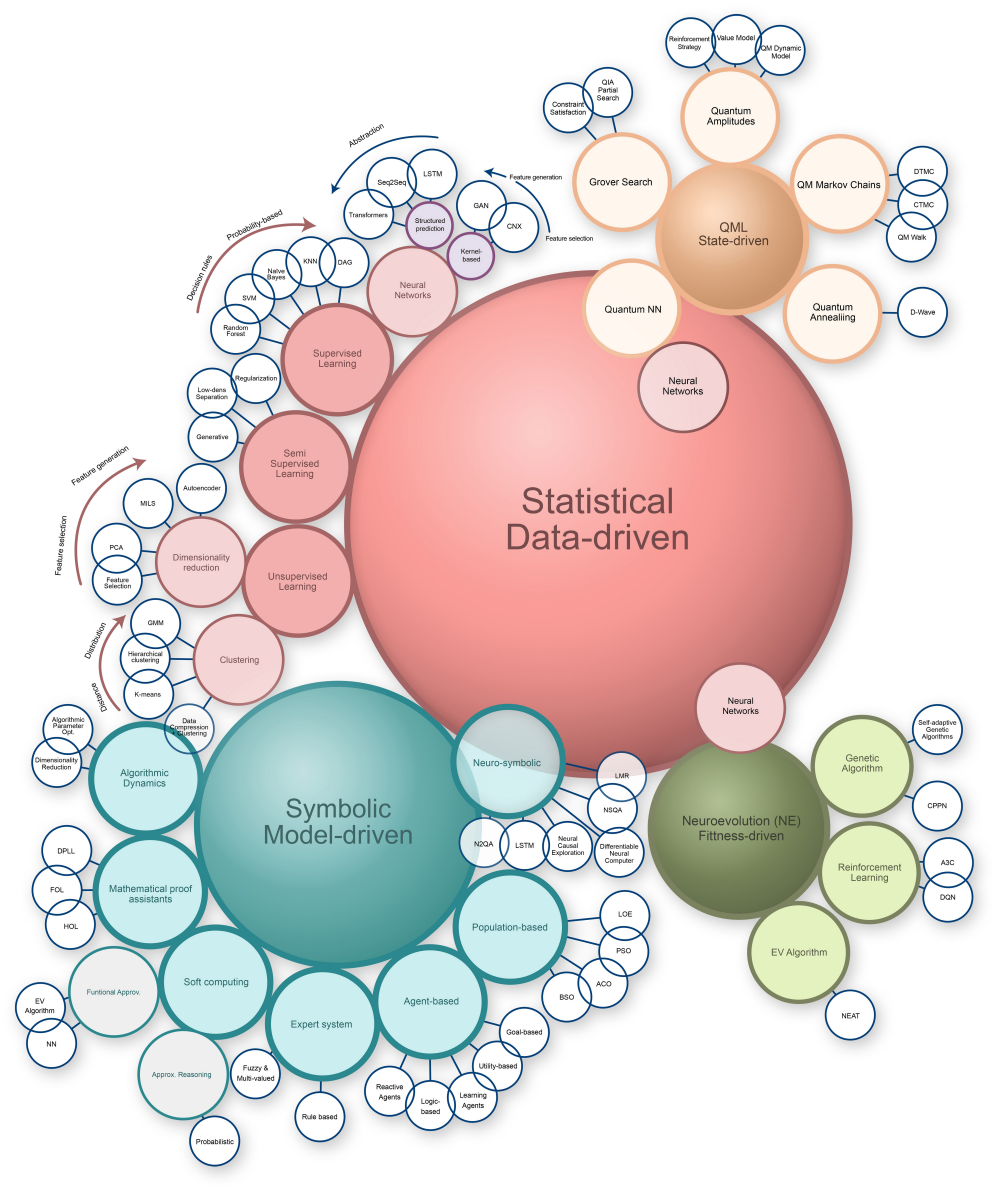
Bubble landscape of current approaches to AI from and for science. Bubbles may occur more than once when related to several larger domains. Some approaches may have alternative names or have been re-branded in certain contexts. Neurosymbolic models have sometimes been referred to as intuitive, while some statistical-driven approaches have been labeled as cognitive computing. Generative AI (GenAI) has made limited contributions to fundamental science so far but has significant potential. Large Language Models (LLMs) may profoundly expand the exploratory range of the scientific hypothesis space, given their ability to process the linguistic corpus where all human science is expressed. GenAI and LLMs remain statistical in nature, though the extent to which they might develop symbolic reasoning capabilities from statistical patterns remains an open question.

In Figure 2, we have mapped subjectively a large number of AI architectures into their Symbolic or Statistical groups and how the interact and divide. Each bubble has evolved and continue to evolve, some disappearing and other emerging in an ever-changing AI landscape.

## 3 Aspects of AI-led closed-loop science

The ability to predict and design (inverse design), while exceptionally useful, will not necessarily lead to new fundamental discoveries (new theories) unless AI and human goals in scientific discovery are aligned and synergistically intertwined to impose similar objectives quantified and introduced, for example, a loss function.

**Figure 3:**
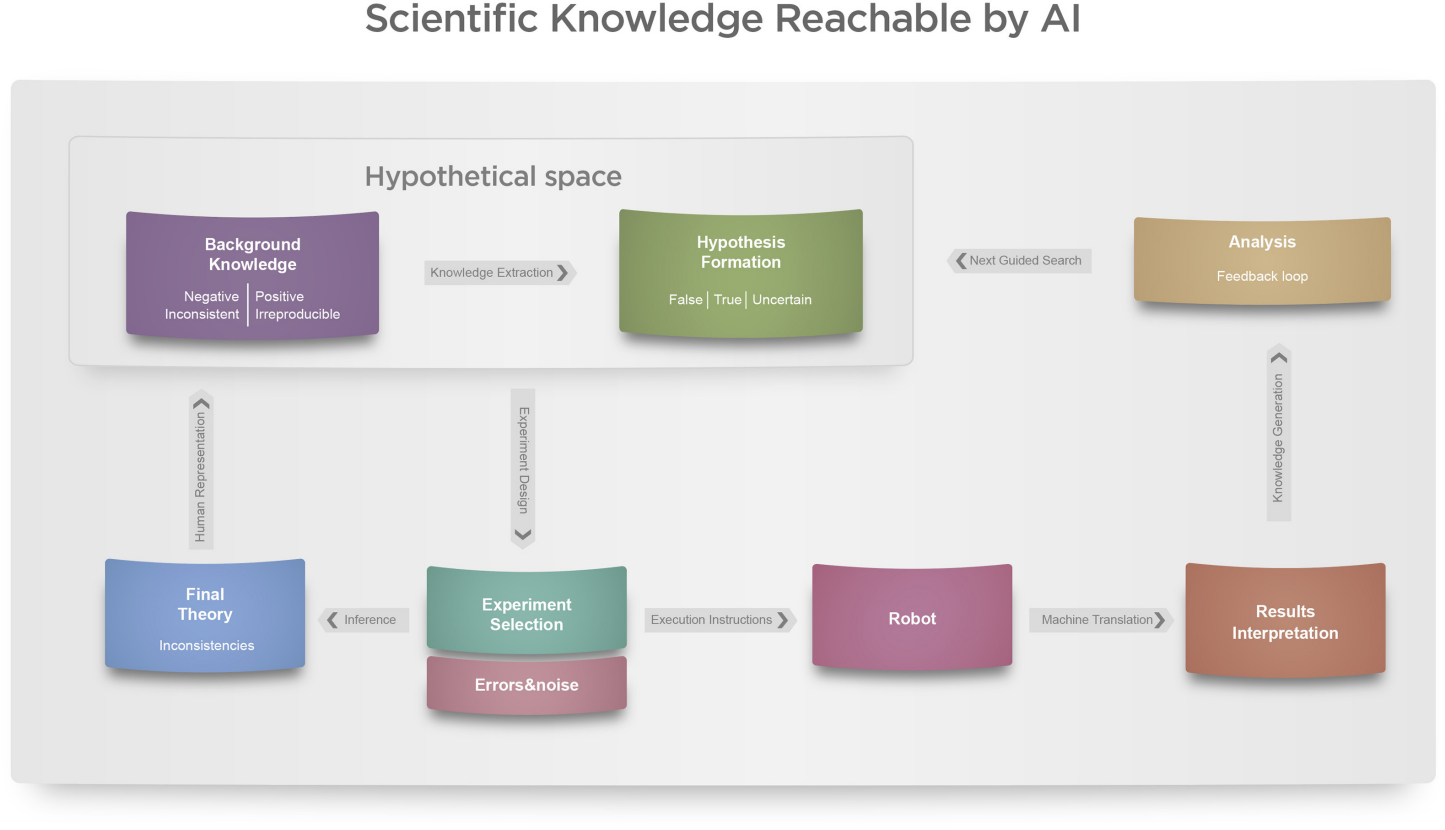
Visual representation of closed-loop full experimentation cycle for scientific discovery pathways, adapted and combining ideas from Kitano (2016) and King et al. (2004). LLMs can now facilitate closing this loop but require help to connect each module and process in a causal rather than only a statistical fashion.

This is because scientific discovery cycles, such as those illustrated in Figure 3, are not isolated parts but belong within a greater cycle of scientific inquiry spanning an entire topic or field comprised of a community of scientists.

It is the larger learning cycle that fuels the questions in the smaller learning cycles. The larger cycle is fuelled by human curiosity and human challenges and has a strong historical and social component, but the shorter cycles, being more well-defined, they are more prone to be automated. Nevertheless, the larger cycles may be needed to kick-start the discovery process of the smaller learning cycles.

In this sense, one option to integrate human scientists and AI-driven science is for humans to build the context of the greater cycle (for example, fulfilling the role of the “Final Theory” and “Background knowledge” steps at the leftmost smaller cycle in Figure 3), feeding the AI with new insights, and leave the AI to independently deal with the smaller cycles (such as the rightmost smaller cycle in Figure 3), guided by the greater ones. An LLM could, for example, be very useful as a technical interface and translation of human high-level larger cycle aspirations and their respective “divide-and-conquer” breakdown into smaller cycles. If one aims at the highest degree of automation of the discovery cycle, more sophisticated forms of AI should include automation of the validation, dissemination, refereeing, and other aspects of human science and its practice.

To tackle such challenges, we propose in the following sections the steps and technology suggested to conduct an entire cycle of AI-led scientific discovery (Gil et al., 2014), as in Figure 3.

### 3.1 Hypothesis generation

One of the central components of the scientific enterprise is the *hypothetico-deductive* method (Popper, 1972; King et al., 2011). Classical epistemology complements deduction with three additional instruments of inference: induction (Bertrand Russell, 1912), abduction (King et al., 2009), and counterfactual reasoning (Pearl, 1995). Together, these provide the cognitive infrastructure through which hypotheses are generated, tested, and revised. To automate such processes, deduction can be integrated with simulation, allowing an AI system to infer the experimental consequences of hypothetical models and to evaluate them against empirical data. Through iterative cycles of experimentation and perturbation (Zenil et al., 2020), the machine can, in principle, arrive at internally consistent theories without direct human intervention.

However, automating hypothesis generation involves far more than clustering, regression, or other inductive procedures. Induction alone merely compresses the regularities already present in the data. The deeper epistemic leap occurs with abduction—the generation of new, causally plausible explanations—and with counterfactual exploration, the construction of alternative worlds that could reveal latent causal mechanisms. Automating these requires a system capable of traversing and expanding its own conceptual space, not just fitting models within an existing one (Abrahão and Zenil, 2022). An AI that merely replays historical patterns, however sophisticated its statistics, cannot exceed the epistemic boundary conditions of its training data.

In this sense, conventional neural networks are poorly suited for genuine hypothesis generation. Their architecture presupposes training data and optimisation objectives, yet in science these are the very objects of discovery. Training on existing hypotheses, classified as valid or rejected, risks reinforcing human biases and foreclosing exploration of unconventional or low-probability regions of the hypothesis space. For this reason, hypothesis generation demands a bottom-up or hybrid approach—one that is model-driven or self-modeling—capable of systematic and potentially unbounded hypothesizing from first principles or universal enumerations, even if such enumeration is redundant or computationally extravagant (Morgan, 1971; Thieme, 2005).

In (Abdel-Rehim et al. 2025), we have proven how LLMs can help produce hypotheses. In this case, drug combinations were tested in the lab for validation purposes with the help of human scientists. The results were fed back into the next finding cycle, hence closing the loop in a first of its kind.

A promising direction in this context is *algorithmic information dynamics* (AID) (Zenil et al., 2020), a framework grounded in combining statistical Machine Learning, causal inference and algorithmic information theory to infer causal generative models. By treating hypotheses as candidate programs that produce observed data, AID searches the algorithmic space for the simplest explanatory mechanism consistent with evidence. This approach allows for abductive reasoning under minimal prior assumptions and can, in principle, generate entirely novel mechanistic hypotheses by exploring the boundary between compressibility and randomness.

Open-ended innovation in hypothesis generation—how to systematically explore unbounded hypothesis spaces in poorly specified domains—remains a frontier problem. These spaces are often non-enumerable or computationally intractable, limited by the same uncomputability constraints that define universal computation (Zenil et al., 2018, 2019a). This uncomputability is not a flaw, but a defining feature of creativity itself: any system capable of generating genuinely new theories must occasionally transcend algorithmic predictability. Navigating such a landscape requires balancing two opposing drives: convergence toward parsimony and divergence toward novelty. Techniques such as partial dovetailing (Lenat, 1982) and bounded search heuristics are needed to avoid infinite regress while maintaining epistemic openness.

Each method for hypothesis generation occupies a position along a continuum between statistical inference and algorithmic construction. At one extreme, statistical methods can guide experiments efficiently by correlating features within ergodic or stochastic processes, though they often conflate correlation and causation (Zenil et al., 2017; Zenil, 2020). At the other extreme, algorithmic frameworks like AID seek generative programs consistent with observations regardless of whether the underlying process is stochastic, computable, or hybrid (Zenil et al., 2018, 2019a). Between these poles lie hybrid approaches—deconvolution algorithms (Zenil et al., 2019b), non-differentiable optimisation schemes (Hernández-Orozco et al., 2021), and open-ended evolutionary computation (Hernández-Orozco et al., 2018a,b)—that attempt to reconcile interpretability with generative power.

A particularly significant advance will be the integration of AID-like causal frameworks with large language models (LLMs). While LLMs remain fundamentally statistical, their ability to represent and traverse symbolic and linguistic structures makes them valuable as epistemic interfaces. They can translate between the high-dimensional formal spaces explored by algorithmic systems and the human-readable representations necessary for understanding and validation. In this configuration, LLMs could serve as a bridge between ‘alien', non-human hypothesis spaces and the interpretive frameworks of human science, allowing us to interact meaningfully with the products of machine-led reasoning.

Looking ahead, the advent of artificial general intelligence (AGI) could further transform hypothesis generation by decoupling it from human cognitive priors altogether. Such systems might explore regions of the conceptual or mathematical landscape that no human has imagined, producing explanations that are empirically consistent yet ontologically foreign. These “alien hypotheses” could represent not failures of comprehension but glimpses of deeper causal architectures inaccessible to human intuition. The critical question will not be whether Artificial General Intelligence (AGI) or Artificial Super Intelligence (ASI) can generate hypotheses, but whether humans can recognize, validate, and integrate them into our own epistemic fabric. In this sense, the automation of hypothesis generation is not merely a technical milestone—it is the point at which science may encounter a truly non-human mode of understanding.

A question related to reaching AGI or ASI with their ever moving fuzzy definitions is how to evaluate progress and capabilities, including “intelligence” in a less anthropocentric fashion. In a recent benchmark that goes beyond the ARC-AGI test that the authors claim mostly evaluates human, similarity in answering spatial questions, a SuperARC test was introduced to evaluate intelligence in terms of prediction and planning, as in model abstraction, based on a test of recursive compression (as opposed to statistical pattern matching) and in contrast to tests designed by and for human intelligence, hence allegedly more agnostic (Hernández-Espinosa et al., 2025b). This is likely a direction that shows promise in evaluating scientific progress beyond human practice that may be bounded by human understanding and human biases, while similar ideas also based on computability and complexity theories can serve as a proxy for AI alignment, the challenge of making AI relevant to human goals and values (Hernández-Espinosa et al., 2025b).

### 3.2 Experimentation and sensing

A central objective for the next generation of AI systems in science is the automation of experimentation and hypothesis testing with minimal or no human intervention. The goal is not merely to design algorithms that execute predefined experiments, but to develop open-ended systems capable of formulating and pursuing their own experimental agendas. Such systems would autonomously select, refine, and generate hypotheses based on the results of prior experiments—whether their own or derived from the accumulated corpus of human knowledge. In this framework, experimentation ceases to be a reactive process and becomes a form of active epistemic exploration.

The integration of sensing, actuation, and reasoning is key to this transformation. To advance beyond the simulation or inference layers of digital science, AI systems must acquire a degree of physical embodiment, enabling them to interact directly with the empirical world. This embodiment closes the lower-level loop of observation and intervention within the broader AI-led closed-loop of scientific discovery. Robotics thus becomes the material substrate of autonomous experimentation: an AI equipped with robotic agents can translate abstract hypotheses into tangible actions, execute them with sub-millimetric precision, and measure their outcomes with reproducible accuracy (Kitano et al., 1997). As robots work faster, longer, and with greater consistency than human scientists, they can exponentially increase the rate and reliability of empirical data generation.

However, embodiment is not simply a matter of mechanical precision. Without it, experimentation risks degenerating into data analysis—an exercise in inference unanchored from the empirical world. Theories and models require feedback from observation and manipulation to maintain epistemic validity. The act of measurement is itself a form of knowledge creation, constraining the space of viable hypotheses. An AI scientist deprived of sensory or instrumental embodiment would therefore operate within a self-referential data loop, potentially leading to epistemic drift. Physical interaction—through controlled perturbation, measurement, and recalibration—is essential to prevent this collapse and to preserve the experimental foundation of scientific reasoning.

Neural networks can assist in embedding physical systems within perceptual and representational frameworks. Their capacity to map high-dimensional sensor data, identify visual and acoustic regularities, and infer spatial relations makes them ideal for interpreting experimental outcomes and controlling robotic systems in real time. Yet, such representational competence must be complemented by advances in robotics, mechatronics, and precision instrumentation. Only through integration of perception, manipulation, and reasoning can AI scientists conduct the full spectrum of experimental activities—from pipetting and microscopy to spectrometry and environmental monitoring—with the rigor and reproducibility required for high-quality science (Baker, 2016). The automation of experimental procedures is not only a question of efficiency but of semantic precision: reproducibility improves when the instructions, actions, and outcomes of experiments are represented within a unified formal language that machines can interpret and execute deterministically.

Large language models (LLMs) will play a critical mediating role in this transition. Their ability to translate between natural and formal languages allows human scientists to specify experimental protocols in ordinary language, which can then be automatically parsed, verified, and executed by robotic platforms. Conversely, results generated by autonomous laboratories can be expressed in human-interpretable form, creating a bidirectional interface between symbolic reasoning and empirical action. The LLM thus functions as an epistemic interpreter, aligning human conceptual frameworks with the operational logic of autonomous experimental systems.

Looking ahead, the emergence of more general AI systems may redefine what it means to experiment. Artificial general intelligence (AGI) could design experiments not simply to test predefined hypotheses but to explore previously unimagined domains of empirical possibility—regions of parameter space that human intuition would never traverse. Such systems might discover novel experimental observables or engineer entirely new measurement modalities, expanding the very boundaries of what can be sensed or known. This would constitute a qualitative shift from automation to epistemic agency: machines not only performing science but actively redefining its ontological reach. The challenge will be to ensure that this expansion remains grounded in physical reality while retaining interpretive accessibility, so that the resulting science, however alien in its methods or insights, remains integrated within the human continuum of understanding.

### 3.3 Rejection, validation and model selection

In scientific reasoning, rejection and validation are not end-points but integral parts of a recursive epistemic process. Model selection represents the bridge between hypothesis generation and the consolidation of scientific knowledge, determining which representations survive the cycle of empirical refutation and theoretical pruning. The problem is ancient yet newly transformed by AI. Machines can now evaluate models not only by fit to data but by the causal or algorithmic efficiency with which those models explain observations.

Model selection and reduction have long been central in fields such as computational biology and neuroscience, where nonlinear systems with multiple time scales and feedback loops require simplified representations for interpretability. A classic example is the reduction of the four-dimensional Hodgkin-Huxley model to the two-dimensional FitzHugh-Nagumo system (Lindner and Schimansky-Geier, 1999). Through time-scale separation, this reduction preserved essential dynamics while revealing which parameters and state variables were most responsible for emergent behavior. Analogous principles apply in AI-driven model compression: identifying minimal sufficient representations that retain explanatory power. Techniques for dimension reduction and feature selection—from principal component analysis to minimal information loss estimators—are instrumental at this stage.

In automated science, the same logic extends into active hypothesis testing. Each hypothesis can be assigned a posterior probability of correctness together with an associated cost of validation, which may include monetary and temporal penalties. The aim is to allocate experimental resources such that expected epistemic gain is maximized under these constraints. Bayesian experimental design provides a rigorous formalism for this, and empirical studies confirm that algorithmic selection of experiments can outperform human intuition (King et al., 2004). Closed-loop implementations can thus operate as self-optimizing inference engines, continuously refining their priors from feedback to converge toward parsimonious, high-yield models.

Current AI systems are already competent at coping with noisy, incomplete, and heterogeneous data, extracting latent structure where human inspection would fail. Neural networks have proven capable of isolating robust signals across physics and biology (Drton and Maathuis, 2017; Eddy, 2004), while optimisation techniques—such as multi-armed bandit methods—can reduce experimental expenditure by adaptively refining sampling strategies. Graphical structure-learning methods (Drton and Maathuis, 2017) provide another layer, enabling machines to infer statistically meaningful dependencies that serve as scaffolds for mechanistic modeling.

Yet the philosophical question remains: what constitutes “validation” when the evaluating agent is itself non-human? In closed-loop systems, the same AI that generates a hypothesis may also judge its success, potentially collapsing the distinction between theory and experiment. One safeguard is to impose multi-level validation: a machine proposes, tests, and selects models, while higher-order algorithms or human scientists verify that these models remain interpretable and epistemically coherent. In this sense, validation becomes a meta-scientific process distributed across layers of autonomy, where machine-led refutation cycles extend the hypothetico-deductive method into the realm of continuous self-correction.

Ultimately, model selection in AI-driven science is not merely an exercise in optimisation but a redefinition of what it means for a model to “explain”. As hypothesis testing becomes automated and iterative, explanation itself becomes procedural—a function of recursive refinement rather than static comprehension. The challenge for the coming era of machine-led discovery will be to ensure that the criteria of rejection, parsimony, and explanatory sufficiency remain anchored to the principles of science rather than collapsing into opaque algorithmic performance metrics.

### 3.4 Knowledge representation and natural language processing

Knowledge representation defines the epistemic substrate of any scientific intelligence. For AI-led discovery, the ability to encode, retrieve, and reinterpret scientific information determines both the efficiency of exploration and the interpretability of results. Large language models (LLMs) have emerged as a pivotal technology in this regard: they can parse and synthesize the corpus of human scientific literature, expressing connections between ideas across disciplinary boundaries. Yet they also introduce a paradox. The same statistical abstractions that make LLMs fluent can conceal deep misalignments between linguistic coherence and semantic truth, complicating traceability, validation, and accountability.

To operate as autonomous scientists, AI systems must integrate rule-based, probabilistic, and linguistic forms of representation. The ingestion of knowledge expressed in natural language requires the system not only to read but to reconcile contradictions, evaluate evidential weight, and identify conceptual gaps within the literature. This implies that the LLM must become self-explanatory—capable of exposing the reasoning pathways underlying its outputs, even when those do not directly mirror its internal statistical derivations. Complementary mechanistic models, grounded in causal reasoning or algorithmic information dynamics (Zenil et al., 2020), may therefore be needed to verify or interpret the outputs of linguistic systems whose inferences are not explicitly computable.

Without such interfaces, knowledge integration would require exhaustive database curation—an infeasible task for most scientific domains. Some areas, such as molecular biology, have advanced ontological infrastructures (e.g., the Gene Ontology, Human Phenotype Ontology, Ontology of Biomedical Investigation) (Stevens et al., 2000; Bard and Rhee, 2004; Shefchek et al., 2020), while others remain far less structured. Initiatives such as the JST MIRAI “Robotic Biology” project have begun to bridge this gap by developing common languages for laboratory protocols (LabCode) and automated IoT-driven experimental workflows. These represent early forms of computable experimental semantics.

At a higher level, probabilistic logic frameworks such as statistical relational learning (SRL) (Raedt, 2008) and relational learning (RL) provide the expressive power to encode not just data but general scientific principles, enabling abstraction beyond individual instances. These methods can incorporate background scientific knowledge while remaining open to revision. When coupled with causal inference formalisms such as the *do*-calculus (Pearl, 1995, 2012) or algorithmic causal analysis (Zenil et al., 2020), they can connect symbolic representation with physical reasoning, creating a continuum between language, logic, and mechanism.

Deep neural architectures complement these efforts by mapping phenomena into high-dimensional vector spaces where structure and dynamics can be represented compactly. Their success in protein folding (Wei, 2019; Jumper et al., 2021) illustrates the potential of purely data-driven encodings, though their opacity raises epistemological concerns. For instance, when state variables or features—such as cytokine networks or drug docking dynamics—are selected automatically (Tang et al., 2019; Zenil et al., 2017), the resulting models may be formally predictive yet semantically inscrutable. High-dimensional embeddings can distort information content exponentially with increasing dimensionality (Abrahão et al., 2021), limiting interpretability and reproducibility.

The future of knowledge representation will thus require hybrid systems combining symbolic and sub-symbolic components, human- and machine-readable semantics, and context-aware translation between them. AI must not only represent scientific knowledge but also communicate it: producing human-understandable narratives, publications, or visualizations of discoveries. This demands a new generation of explainable translators—LLMs aligned with causal, algorithmic, and ontological frameworks—to ensure that meaning survives the transition between human and machine cognition.

Such advances will extend beyond representation to meta-representation: encoding not only facts about the physical world but also the dynamics of the scientific process itself. Capturing the social and procedural interactions among human and machine agents (Evans and Foster, 2011) could allow future AI systems to model the evolution of science as a complex adaptive network. These capabilities, when fully realized, would not only mitigate issues of reproducibility and bias but also redefine the epistemic infrastructure of discovery. In this view, the evolution of scientific language, human or machine, becomes inseparable from the evolution of science itself.

### 3.5 Integration, interpretation and interfacing

Integrating new information into the corpus of existing scientific knowledge remains one of the most intellectually demanding aspects of discovery. The challenge is not simply to append new data to old, but to reconcile, reinterpret, and sometimes overturn the conceptual scaffolding that holds prior theories together. In the human tradition of science, this task has relied on judgment, intuition, and serendipity—the capacity to see significance in apparent anomalies. In the context of AI-led discovery, such reinterpretation must itself become algorithmic. Machine learning systems must not only detect patterns in data but also identify contexts in which rejected or marginal hypotheses acquire new explanatory relevance.

This process corresponds to what might be called the larger loop of learning, in contrast to the narrow loops of hypothesis testing and experimentation. A system capable of operating in this broader context must continuously evaluate whether its findings are consistent with or orthogonal to established knowledge, adjusting its internal representations accordingly. Serendipity, in this sense, becomes an emergent property of algorithmic reinterpretation: when results obtained for one goal become significant under a different model or ontology. Such systems will require both high-level abstraction and fine-grained contextual reasoning, linking local experimental outcomes to global scientific narratives.

Machine learning already contributes to automated knowledge integration at scale. Text-mining and information-extraction algorithms have been used to construct vast knowledge bases from the scientific literature, such as genome-wide association networks and drug-disease interaction databases (Andronis et al., 2011). These systems accelerate discovery by ensuring that new results are rapidly situated within the existing body of knowledge, allowing unexpected correlations to be detected across disciplines. However, genuine understanding requires more than accumulation; it demands interpretation—an awareness of the semantic, social, and epistemic structures that govern how knowledge is produced and used (Fortunato et al., 2018).

AI systems must therefore be situated within knowledge-rich, multi-agent environments (Kitano et al., 1997), capable of modeling not only scientific content but also the social and cognitive dynamics of the scientific community. The integration process becomes a negotiation between human and machine agents, each contributing complementary strengths: human interpretative depth and ethical awareness, and machine scalability, speed, and memory. This coupled system must be optimized holistically, treating human and AI scientists as components of a single evolving epistemic organism. The objective is no longer to replace human reasoning, but to co-evolve with it.

A useful analogy can be drawn from the evolution of human-machine collaboration in chess. Following Garry Kasparov's defeat by Deep Blue, the most successful chess players became those who integrated computer assistance into their own strategy (Campbell et al., 2002). Today, the highest-performing freestyle teams are not composed solely of humans or machines, but of ensembles that exploit the unique reasoning capabilities of each. Similarly, the future of science may belong not to autonomous AI or isolated human investigators, but to integrated collectives that jointly navigate the expanding frontier of the hypothesis space.

Achieving such integration will require AI architectures that combine statistical learning with symbolic inference, allowing knowledge to remain both computationally actionable and humanly intelligible. Classical logical inference engines (McCarthy, 2007) will be essential for interpretability, while neural and probabilistic systems provide scalability and adaptability. A meta-analytic layer capable of simulating the network topology of scientific production (Dashun and Albert-László, 2021) could enable these hybrid systems to monitor the structure, biases, and emergent properties of science itself, adjusting their own behavior to maintain diversity and epistemic balance.

The advantages of this co-evolutionary model are substantial: reduction of human bias, improved reproducibility, and acceleration of cross-domain synthesis. However, these same features introduce new challenges of verifiability and transparency. A coupled human-AI system must be formally auditable to ensure alignment between process and outcome (Hernández-Espinosa et al., 2025a). Both agents must remain capable of explaining and justifying their reasoning, even when operating on different cognitive or computational scales. Designing such systems will require AI algorithms to continuously reinterpret not only their data but their role within the broader epistemic system, learning from the outputs and feedback of their human collaborators. In turn, human scientists must refine their interpretative and analytical skills to engage critically with machine-generated results. Together, these dynamics will redefine not only the practice of science but the nature of scientific explanation itself.

### 3.6 Closing the loop

The completion of the closed-loop cycle in AI-led scientific discovery represents the convergence of all preceding components—hypothesis generation, experimentation, validation, and integration—into a self-regulating meta-system. To achieve this, a higher-order algorithmic layer must orchestrate and monitor the iterative cycles of inquiry, deciding when to continue, restart, or terminate them (see Figure 3). This supervisory mechanism functions as the metacognitive component of the artificial scientist: an agent that observes and optimizes its own processes of discovery.

Human intervention must remain an integral part of this architecture, not as a bottleneck but as an epistemic checkpoint. The loop should be permeable to human feedback, allowing scientists to contribute insights, reinterpretations, or ethical constraints, while enabling the AI to correct for human biases or limitations in scope. Such bidirectional oversight ensures that the trajectory of machine-led science remains both autonomous and aligned.

From an engineering perspective, closing the loop requires robust infrastructural and computational systems. Remote, web-based control of scientific instrumentation may depend on frameworks such as TypeScript, React, GraphQL, Jest, and Redux, which enable scalable interfaces between experiment automation, data processing, and analytical oversight. Optimisation and anomaly detection algorithms play critical roles: they identify discrepancies or gaps that may signal either methodological errors or opportunities for new discovery. In this way, the AI system can detect the unexpected—reinterpreting anomalies as potential starting points for new hypotheses, thereby reopening the cycle of exploration.

The grand challenge (Kitano et al., 1998) of fully automated, AI-led science is therefore not only to execute experiments or infer models, but to institutionalize a complete epistemic pipeline—from data acquisition to publication, from theory building to peer review. Each component of this pipeline must be interoperable, transparent, and reflexive. Closing the loop does not signify the end of human science, but rather its transformation into a collaborative ecology of intelligences. The ultimate aim is not to mechanize discovery but to universalise it—to create systems capable of continuous, self-correcting inquiry that expand the reach of human understanding while preserving its meaning.

## 4 Conclusions

Future scientific progress has become almost inconceivable without the participation of artificial intelligence. Machine learning now permeates every stage of scientific inquiry, from hypothesis generation and experiment design to interpretation and dissemination. Yet, as we have argued, the real transformation lies not in automation alone but in the emergence of closed-loop systems that complete the experimental cycle without requiring human intervention to initiate the next iteration. Such systems represent an epistemological shift: they promise not only to augment and accelerate discovery but also to reshape the trajectory of science itself.

Closed-loop architectures will likely define the next epoch of the scientific method. By continuously linking observation, hypothesis generation, experimentation, and evaluation, they create the conditions for self-correcting scientific reasoning operating at machine timescales. When implemented with sufficient safeguards, these systems could mitigate entrenched human biases, reduce error propagation, and enhance reproducibility across domains. In doing so, they do not just accelerate science but potentially make it more objective: they balance the human contribution from manual labor and intuition to conceptual framing, ethical oversight, and interpretive synthesis. The risk, however, is that these systems may also diverge from human intuitions about explanation and meaning, producing models that are empirically powerful but conceptually opaque.

The application of AI in scientific discovery presents us with very different challenges compared to the application of AI to games such as chess, shogi, or Go. However, recent developments suggest that creativity may come from two possible sources, and not too different from games (Silver et al., 2016; Hassabis, 2017; Kitano, 2021), in particular chess. On the one side, new strategies that may or may not be understood by humans. But also an aspect of playing under time constraint and the gain of speed that AI-driven discovery may provide. While we have argued that this may be domain specific, AI alone may offer an unmatched acceleration compared to both human-led science and even human-AI-interaction-driven science because of additive constants and misalignment.

The analogy with strategic innovation in chess illustrates this tension. Magnus Carlsen's adoption of AlphaZero-inspired play exemplifies a transition where a human expert exploits algorithmically emergent strategies that are demonstrably superior yet only partially comprehensible. Similarly, future scientists may find themselves collaborating with systems whose inferences they can validate but no longer fully rationalize. The closed-loop scientist, human or artificial, will thus operate at the boundary between comprehension and performance, exploiting correlations that work even when their causal or theoretical underpinnings remain elusive.

In this light, the role of the scientist is poised to evolve. The future of scientific inquiry will depend less on the direct manipulation of data and more on the governance of epistemic processes—deciding when and where to trust, interpret, or contest machine inference. Graded autonomy will become central: domains such as materials science or molecular biology, with rapid experimental feedback, may lend themselves to near-total automation, while fields requiring interpretative synthesis, such as medicine or the social sciences, will remain human-anchored. The challenge is to determine when the interaction cost *C*_int_ (on both sides of the equation) between humans and machines turns from synergy to friction, and to design hybrid systems where *T*_*H*+*M*_ < min(*T*_*H*_, *T*_*M*_) increases not only in speed but also in explanatory yield. That is, whether there is a deep integration of human and machine that produces more and better science across all domains. What we have suggested here is that this will be domain-specific and will evolve over time.

This transformation also compels us to revisit the aims of science itself. If AI can autonomously explore vast regions of the hypothesis space, the frontier of discovery may begin to advance in directions that no human has defined or anticipated. The resulting discoveries might be valid, verifiable and reproducible but alien to our conceptual vocabulary and perhaps strangled from human understanding. The epistemic singularity—when the rate and depth of AI-led discovery exceed human comprehension—may not appear as a discrete event but as a gradual decoupling between explanation and understanding. Whether this marks an end or an expansion of science depends on how we design the interfaces that mediate between human cognition and machine reasoning.

On the one hand, new questions now confront both scientists and policy makers. Should agentic AI systems be endowed with sufficient autonomy to pose their own questions and lead their own goals? How do we ensure that their objectives remain aligned with human priorities (Hernández-Espinosa et al., 2025a)? Who decides which domains are safe for unsupervised exploration and who bears responsibility for the discoveries that follow? These are not speculative concerns: they shape the way AI4Science is governed, shared, and trusted today.

On the other hand, the stakes are too high to delay. We face global challenges: social and political polarization, climate change, antibiotic resistance, which demand an order-of-magnitude increase in our capacity for hypothesis-driven experimentation. Humans alone cannot meet this demand. The integration of AI into the closed loop of science is therefore not optional but necessary. The crucial task ahead is to build systems that accelerate discovery without severing the link between knowledge and meaning, ensuring that as we hand over the instruments of inquiry, we do not lose sight of the very questions that make science a human endeavor.

Areas of science will be so much more sophisticated than they are today that new challenges and directions are likely to emerge, unrelated to today's ones, so we need to start thinking as a community with our collective mind of what we know we do not know and we won't know, in order to anticipate the next AI-direction move.
